# Fructose affecting morphology and inducing β-fructofuranosidases in *Penicillium janczewskii*

**DOI:** 10.1186/s40064-015-1298-7

**Published:** 2015-09-09

**Authors:** Rosemeire A. B. Pessoni, Carla C. Tersarotto, Cássia A. P. Mateus, Juliana K. Zerlin, Kelly Simões, Rita de Cássia L. Figueiredo-Ribeiro, Márcia R. Braga

**Affiliations:** Faculdade da Saúde, Curso de Ciências Biológicas, Universidade Metodista de São Paulo, São Bernardo do Campo, SP Brazil; Núcleo de Pesquisa em Fisiologia e Bioquímica, Instituto de Botânica, CP 68041, São Paulo, SP CEP 04045-972 Brazil

**Keywords:** Cell wall, Filamentous fungus, Fructose, Glucose, Invertase, Inulinase, *Penicillium janczewskii*, Sucrose

## Abstract

Fructose, glucose, and an equimolar mixture of both sugars affected differently hyphae thickness, biomass production and secretion of β-fructofuranosidase in *Penicillium janczewskii.* Reduced growth, thinner hyphae and visible injuries were early observed during fungal cultivation in fructose-containing medium, reaching the maximum between 12 and 15 days of culture. Total sugar content from the cell wall was lower when fructose was supplied and polysaccharides lower than 10 kDa predominated, regardless the culture age. Maximal inulinase and invertase activities were detected in culture filtrates after 12 days, excepting in the glucose-containing medium. Structural changes in cell walls coincided with the increase of extracellular enzyme activity in the fructose-containing medium. The fragility of the hyphae might be related with both low carbohydrate content and predominance of low molecular weight glucans in the walls. Data presented here suggest changes in carbohydrate component of the cell walls are induced by the carbon source.

## Background

Two basic morphological units can be distinguished in the vegetative stage of fungal growth, the yeast form (or unicellular) and the hyphal or filamentous one. Growth of filamentous fungi occurs by hyphal tip elongation, and the cell shape is primarily determined by the location and rate of cell wall deposition. The fungal cell wall is a supramolecular structure that determines cell shape, resistant to differences in osmotic pressure between the cytoplasm and external environment, and provides protection against mechanical damage (Ruiz-Herrera et al. 2008). The cell walls consist mainly of carbohydrates and free and bound proteins (Santos et al. 2000). The main components of fungal cell wall are β-1,3-d-glucans, which can contain branches with glucose residues β-1,6 connected, and chitin, a polymer of *N*-acetylglucosamine (Santos et al. 2000). Proteins of the cell wall are mainly associated with hetero- or homopolymers of mannose (Fontaine et al. 1997).

The biosynthesis of cell wall is a key process in the growth and morphogenesis of the fungal cells. However, despite their key role in the development of these organisms, little is known about cell wall structure, mainly with respect to the arrangement of various polymers and their effects on the physical and biological properties of the cell wall. Indeed, it is not yet clear how these basic processes are coordinated to produce all the morphological diversity found in filamentous fungi (Latgé 2010). The growth and different metabolic activities of fungi are usually a response to physical–chemical conditions of the environment that surrounds them. The fungi depend on certain elements or compounds present in the environment. Ruiz-Herrera (1992) observed that factors such as nutrition, temperature and incubation time cause significant differences in fungal cell walls. The same was also observed in *Penicillium janczewskii* that grows rapidly on medium containing sucrose or inulin as carbon sources, but only in inulin-containing medium large amounts of inulinases (2,1-β-d-fructan:fructan hydrolase EC 3.2.1.7) were released, which was associated with thin cell walls (Pessoni et al. 2005). Considering that inulin is a polymer of fructose, it was hypothesized that high fructose content in the medium as a result of the released extracellular inulinases could be involved in the morphological changes observed in the hyphae cell walls. In the present work we analyzed changes in cell wall structure and composition of *P. janczewskii* growing on fructose, glucose or fructose + glucose as carbon sources, aiming at a better understanding of the effects of fructose on cell wall structure and composition and production of extracellular β-fructofuranosidases (β-d-fructofuranoside fructohydrolase EC 3.2.1.26).

## Results

### Fungal growth curve and induction of β-fructofuranosidase

**Fig. 1:**
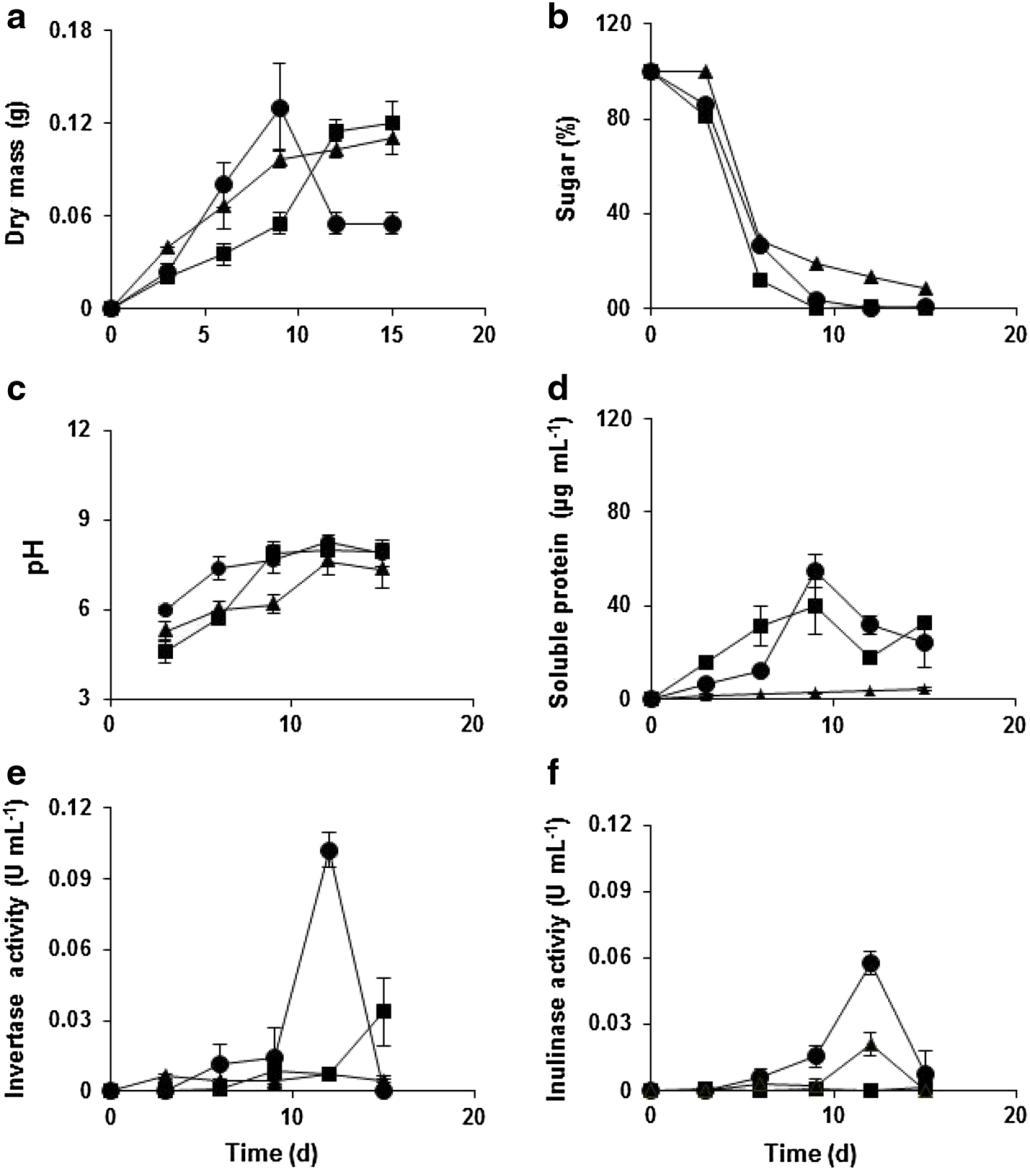
Culture of *P. janczewskii* in liquid medium containing different carbon sources. Dry mass (**a**), consumption of sugars (**b**), pH of the medium (**c**), protein content (**d**), invertase activity (**e**) and inulinase activity (**f**). Fructose (*circle*), glucose (*square*), glucose + fructose (*triangle*)

The growth of *P. janczewskii* was monitored by the increase of the mycelium dry mass and consumption of sugars throughout 15 days of cultivation in different culture media (Fig. 1). The fungus showed an exponential growth phase until day 9, followed by a stationary phase of growth by day 15 in media containing only glucose or glucose + fructose. In the fructose-containing medium a sharp decline in growth was observed from the day 9 on (Fig. 1a). The decrease in the sugar content of the medium coincided with the increase in biomass and was no longer detected from day 9 (Fig. 1b). The amount of protein released by the fungus grown in media containing glucose or fructose was similar (Fig. 1d). The protein content increased up to 9 days, declining after that. Conversely, when the fungus was grown in a mixture of glucose + fructose it was observed a slight increase of proteins throughout the growing period analyzed, but the content was about 10 times lower than those found in the other media.

Different carbon sources distinctly induced the secretion of β-fructofuranosidases. In the glucose-containing medium was observed an increase in invertase (β-d-fructofuranoside fructohydrolase, EC 3.2.1.26) activity at 15 days of culture and negligible activity of inulinase. On the other hand, fructose was effective in inducing the activity of invertases and inulinases, the latter activity being half that observed for invertase. Both activities were higher at 12 days of cultivation. In the medium containing a mixture of glucose + fructose was observed increase of inulinase from day 9, which peaked around day 12, declining after that (Fig. 1f). The current data confirm that the presence of fructose in the medium induces secretion of β-fructofuranosidases (Fig. 1e, f). The pH was similar in different media, increasing along the growth curve (Fig. 1c).

### Growth on solid medium and structural analysis

**Fig. 2:**
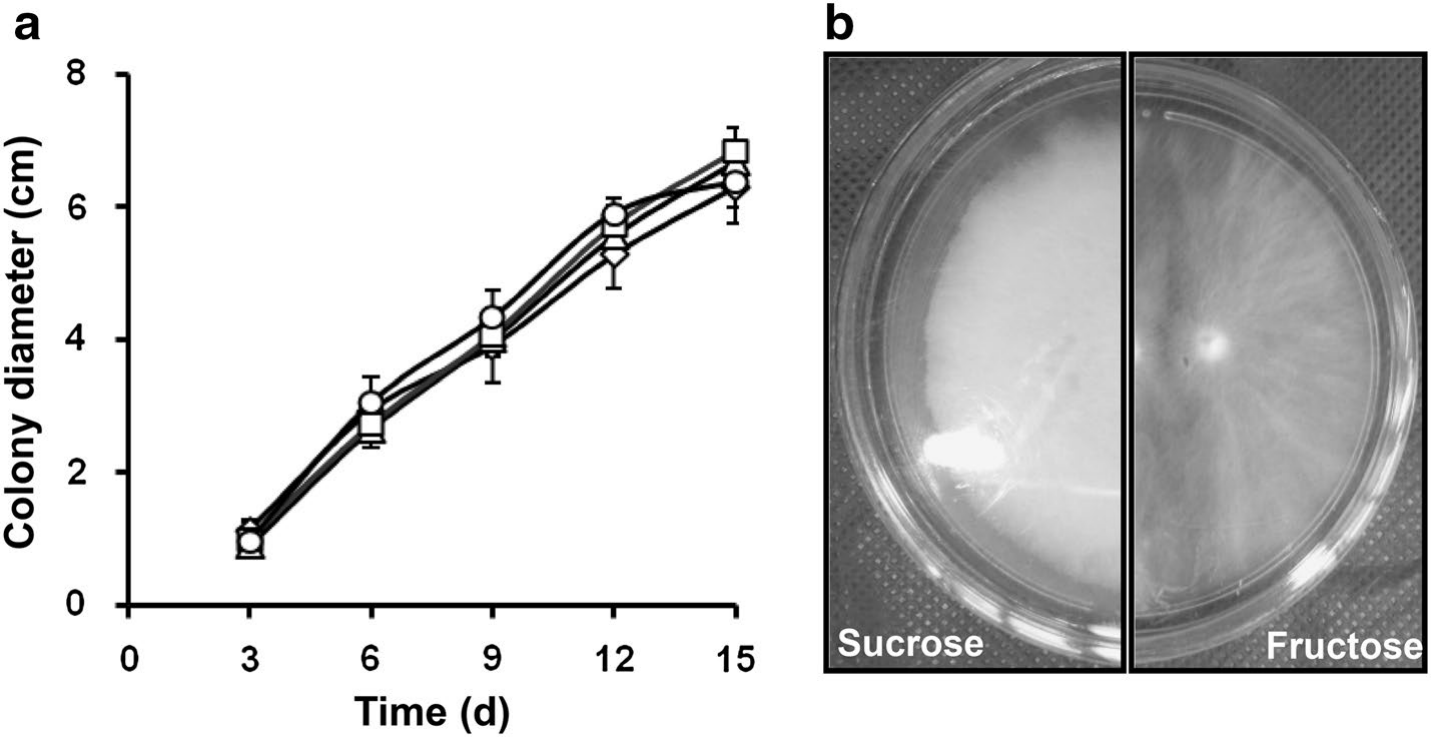
Growth curve of *P. janczewskii* maintained in solid medium containing different carbon sources (**a**). Fructose medium (*circle*), glucose (*square*), glucose + fructose (*triangle*) and sucrose (control) (*diamond*), general aspects of *P. janczewskii* colony grown for 9 days on solid medium (**b**) containing sucrose or fructose as a carbon source

The growth rate of the fungus cultured on solid medium containing different carbon sources was similar (Fig. 2a) but the macroscopic characteristics of the colonies were clearly different (Fig. 2b).

**Fig. 3:**
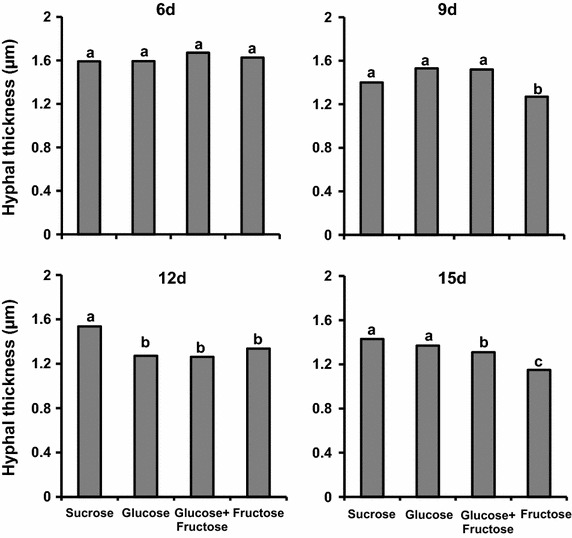
Hyphae thickness (µm) of *P. janczewskii* grown for 6 (6d), 9 (9d), 12 (12d) and 15 (15d) days in medium containing different carbon sources. *Different letters* indicate a statistically significant difference between the groups (*p* < 0.05)

The mycelium grown on fructose was less vigorous and sparser compared with that grew on sucrose (Fig. 2b). Micro-cultivation also showed significant differences in the hyphae thickness, mainly from day 9 of cultivation on (Fig. 3).

In this period, hyphae grown in medium containing fructose was significantly thinner compared to those grown in the other carbon sources. Conversely, the hyphae thickness from medium containing sucrose was significantly higher than that observed in other culture media (Fig. 3).

**Fig. 4:**
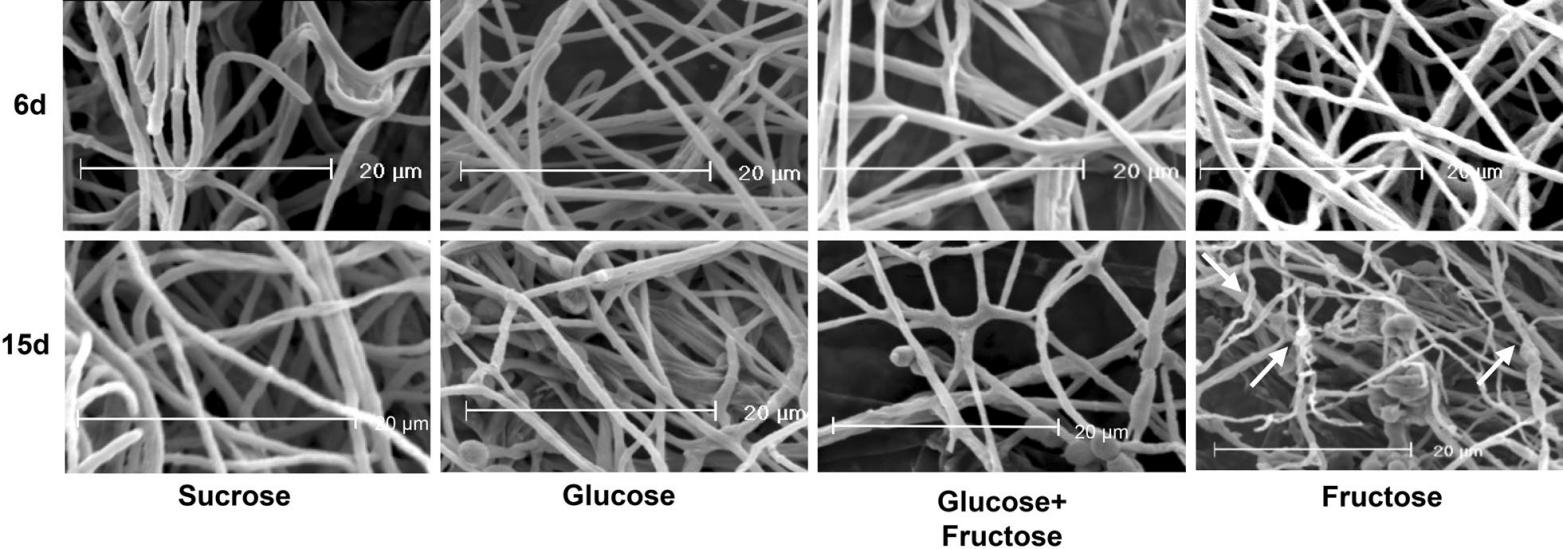
Scanning electron microscopy (SEM) of *P. janczewskii* mycelium grown in different carbon sources. Increased ×1600. *Arrows* indicate the injured and collapsed hyphae

The mycelium was also analyzed by SEM (Fig. 4) and showed morphological changes induced by the carbon source. The hyphae of the mycelium grown in glucose-containing medium was more turgid and no injuries were observed throughout cultivation at 6, 9, 12 and 15 days. On sucrose, collapsed hyphae were observed only after 15 days of growth. Conversely, in the medium containing an equimolar mixture of fructose + glucose, hyphae with constrictions were observed on day 9 and injured and collapsed hyphae were observed from day 12 on. In fructose-containing medium, injured and collapsed hyphae were observed at day 9, and these changes became more conspicuous at 12 and 15 days, which may be indicative of changes caused by the carbon source, particularly by fructose that increased in the medium (Fig. 4). These changes are consistent with what was observed by light microscopy revealing hyphae significantly thinner at day 9 in fructose-containing medium when compared to those cultured in other carbon sources (Fig. 3).

### Characterization of cell wall

**Fig. 5:**
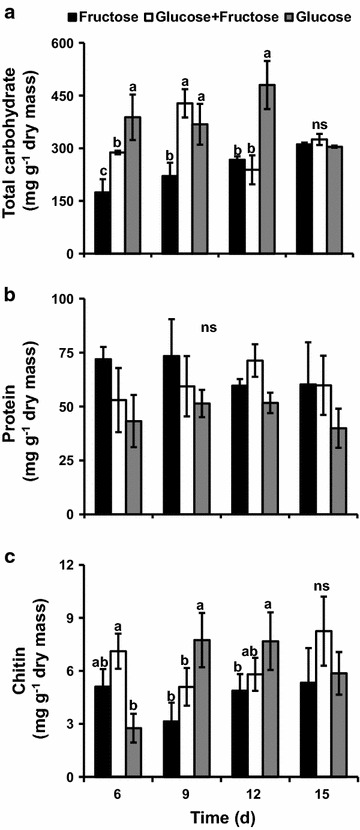
Characterization of the cell walls of *P. janczewskii* grown in different carbon sources. Sugar content (**a**), protein (**b**) and chitin (**c**) in the cell wall. Data represent the average of three replications

The content of sugars, proteins and chitin were determined after hydrolysis of the cell walls (Fig. 5).

The concentration of total sugars in the cell wall of the fungus grown in fructose was significantly lower compared to that grown in medium containing glucose or glucose + fructose as carbon sources. The sugar content of the fungal cell wall decreased with culture age. On the 15th day, no significant differences were detected in sugar content among the different culture media (Fig. 5a).

The protein content did not vary significantly with culture age or carbon source (Fig. 5b) while chitin content tended to increase with the age of the culture in the medium containing glucose as carbon source (Fig. 5c).

**Fig. 6:**
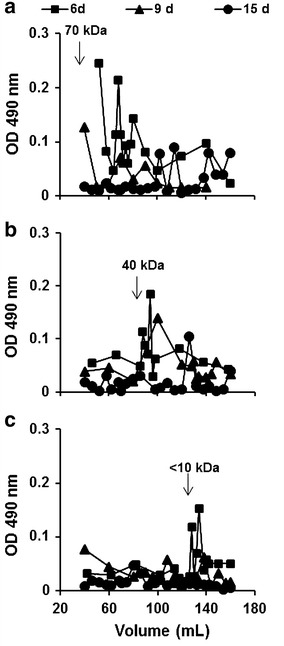
Molecular sieve chromatography—sepharose CL-6B. Elution profile of cell wall polysaccharides from the mycelium of *P. janczewskii* grown in liquid medium containing glucose (**a**) an equimolar mixture of glucose + fructose (**b**) or fructose (**c**) as carbon source

The molecular weight of polysaccharides from the fungal cell wall determined by molecular sieve chromatography varied depending on the carbon source (Fig. 6).

Polysaccharides with higher molecular weight, predominantly between 63 and 10 kDa, were found in the glucose-containing medium at 6 days of culture (Fig. 6a). Conversely, polysaccharides with lower molecular weights were observed in the cell wall of the fungus grown in the fructose-containing medium (Fig. 6a, b). The results obtained so far demonstrated that the cell wall obtained from *P. janczewskii* grown in medium containing fructose showed a decrease in total sugar content (Fig. 5a) and the presence of lower molecular mass glucans (Fig. 6).

## Discussion

### Comparison of growth and induction of β-fructofuranosidase activity of *P. janczewskii* grown in liquid medium using different carbon sources

The growth pattern of *P. janczewskii* cultured in liquid or solid media containing glucose or an equimolar mixture of glucose + fructose (Figs. 1a, 2) was very similar to that previously reported for the same species growing on sucrose or inulin as carbon sources (Pessoni et al. 1999, 2007). The decrease of sugars in the culture medium was accompanied by increased pH values and high content of extracellular proteins, a behavior already reported for other fungi (Chaudhuri et al. 1999; Dhake and Patil 2007). Notably, the pH plays a key role in enzyme production and it is directly related to the enzyme stability. Galeote et al. (2010) reported that proton influx results in alkalinization of the culture medium, suggesting that changes in the pH observed in *P. janczewskii* could be related to the hexose uptake by symporter activity.

The production of extracellular enzymes by *P. janczewskii* was influenced by the carbon source (Fig. 1e, f). The presence of fructose in the culture medium stimulated the release of β-fructofuranosidases as reported previously by Pessoni et al. (2007) for *P. janczewskii* and by Rubio et al. (2003) for *P. glabrum*. According to Romero-Gómez et al. (2000), these enzymes seem to be induced by the carbon source in filamentous fungi. Conversely, catabolic repressor effect induced by glucose and fructose has also been reported in other fungi. In yeast, β-fructofuranosidases are constitutively synthesized, and the presence of glucose or fructose in the culture medium decreases the enzyme activity by gene repression (Costaglioli et al. 1997; Dynesen et al. 1998). In *Aspergillus niger* model, fructose did not induce the expression of inulinolytic genes (Yuan et al. 2006), and in *Penicillium* sp. fructose also failed to induce the expression of inulinases, and glucose exerted a potent carbon catabolite repression (Moriyama et al. 2006). Generally, this repressor effect is associated with increased concentrations of glucose or fructose. In *P. janczewskii* the induction of β-fructofuranosidases occurred only after 12 days of culture, when the sugar levels in the medium were very low, but probably enough to induce the signaling pathway that activates the expression of inulinase and invertases genes. The transcriptional activator InuR was identified as a positive inducer, acting for the induced expression of genes involved in the breakdown of inulin and sucrose, and in the uptake of their products (Yuan et al. 2008). Possibly low levels of glucose or fructose can be involved in the activation of InuR, and consequently in the expression of β-fructofuranosidases (Yuan et al. 2006, 2008).

### Morphology, ultrastructure and cell wall composition of *P. janczewskii* grown on different carbon sources

The maintenance of *P. janczewskii* in the inulin-containing medium induces secretion of large amounts of inulinases but results in hyphae with thin cell walls that easily collapses and breaks; the same was not observed when the fungus was cultured in medium containing sucrose (Pessoni et al. 2005). Although no differences were found in content of sugars and proteins from the cell wall of hyphae grown on these carbon sources, a significant decrease in cell wall thickness was observed when the fungus was grown on inulin (Pessoni et al. 2005). In the present work it was observed that although the growth rate of *P. janczewskii* in solid medium containing fructose was similar to those found in other media (Figs. 1, 2) there was a significant decrease in the thickness of the hyphae (Fig. 3) and in fungal biomass (Fig. 1a). According to Chaudhuri et al. (1999), the increase in fungal biomass is a result of deposition of cell wall polysaccharides during hyphae cell elongation. The changes in the growth parameters of *P. janczewski*i possibly induced by fructose could be related to the significant decrease of total sugar content of the cell walls, observed from day 6 on (Fig. 5), when compared to the glucose-containing medium. Changes in levels of neutral and amino sugars can affect the organization of the cell wall (Ghfir et al. 1997), influencing morphogenesis. SEM analysis of the mycelium of *P. janczewskii* showed changes in the turgidity of the hyphae (Fig. 4) evidencing weak and collapsed hyphae in medium containing fructose, which may be indicative of changes caused by the carbon source.

Chitin and protein, that are structural components of fungal cell walls, also play an important role in morphogenesis and signaling and may change under stressing conditions (Seidl 2008). Damveld et al. (2005) observed a significant increase of cell wall polysaccharides in *A. niger* grown under stressing conditions induced by Calcofluor White. Similarly, de Nobel et al. (2000) reported increased transcription and incorporation of several proteins in the wall as a compensatory mechanism to preserve the integrity of the wall. Levels of protein in the cell walls of *P. janczewskii* were similar regardless of carbon source (Fig. 5). These data suggest that the presence of fructose in the culture medium seemed not to cause a nutritional stress, or at least not a sufficiently sensitive situation to affect the analyzed cell wall components. Liu et al. (2013) demonstrated that changes in chitin synthase genes affected growth rate and hyphae morphology in *Penicillium chrysogenum* enhancing penicillin production. In the present work, although chitin synthase activity was not analyzed, changes in chitin content in the cell walls of *P. janczewskii* suggest a possible effect of the carbon source on such enzymes.

The polysaccharides constitute a significant proportion of fungal biomass. The cell wall of hyphae contains over 75 % of this type of biomolecule, glucans predominating in filamentous fungi (Mellado et al. 2003). Analyses of glucans from cell walls of *P. janczewskii* revealed the presence of polysaccharides of low molecular weight in the mycelium grown in the fructose-containing medium (Fig. 6). According to Silva et al. (2006), most of the polysaccharides are produced directly as responses to environmental factors. For some microorganisms, the carbon source determines the amount of polysaccharide formed, and the quality of the synthesized product. This was also observed in the present work, indicating that the carbohydrate components of the cell wall are responsible for the changes observed in the mycelium of *P. janczewskii*, and that these modifications are induced by the carbon source supplied in the culture medium. According to Santamaría et al. (2002), complex morphological development involves intricate pathways in which intracellular and extracellular signals are perceived by regulatory systems that repress or trigger the process. Among the effectors of these pathways, sugars act not only as nutrients but also as important regulators of gene expression.

The morphology and productivity of filamentous fungi have been extensively investigated, mainly in relation to macroscopic characteristics of the mycelium, with the hyphae formation (Grimm et al. 2005).

The results obtained in this study also indicate that the structural changes in the cell walls occurred when the fungus was cultured in medium containing fructose. The mechanism by which fructose affects the cell wall structure is not clear, but could be related to the biosynthesis of polysaccharides of the cell wall. The first step of carbohydrate metabolism is the uptake of the molecules into the cell. To achieve this, microorganisms employ a variety of different membrane-bound transport proteins. The expression of several of genes of these transport proteins are under transcriptional control depending on the source and the amount of carbon available (Wei et al. 2004; Flipphi et al. 2009).

Fructose-specific uptake in fungi is not common, but has been observed in *Neurospora crassa* when the mycelium was subjected to carbon starvation (Rand and Tatum 1980). Hexose uptake by filamentous fungi such as *Aspergillus nidulans* (Wei et al. 2004) usually occurs with higher affinity to glucose than to fructose. Within the cells, fructose and glucose are phosphorylated by hexokinases (ATP:d-hexose 6-phosphotransferase EC 2.7.1.1) and glucokinases (ATP:d-glucose 6-phosphotransferase EC 2.7.1.2), and the phosphorylated sugars are interconverted to each other by hexose-6-phosphate isomerase. Therefore it is expect that the differential sensing of fructose and glucose occurs before or during phosphorylation (Doehlemann et al. 2005).

In *Lactococcus lactis*, Benthin et al. (1994) reported that when fructose 6-phosphate (Fru-6P) is produced by fructose uptake via constitutive mannose phosphotransferase systems (Man-PTS), both biomass precursors and metabolic energy can be formed. However, when fructose is transported and phosphorylated via the inducible fructose phosphotransferase systems (Fru-PTS), the resulting fructose-1-phosphate (Fru-1P) enters glycolysis as Fru-6P and would therefore have to be a gluconeogenic substrate. In the absence of the biosynthetic pathway leading to fructose 1,6-diphosphate, Fru-1P can be used only for generation of metabolic energy, and certain biomass precursors (Glc-6P or Fru-6P) cannot be formed from Fru-1P. Moreover, fructose-1-phosphate is a powerful inhibitor of the phosphomannose isomerases implicated in glycoprotein processing and biosynthesis (Jaeken et al. 1996) and this can influence the cell wall biosynthesis. These enzymes catalyze the reversible conversion of Fru-6P to mannose-6P during biosynthesis of GDP-mannose, which is the main intermediate in the mannosylation of cell wall components. The interconversion of Man-6-P and Fru-6-P catalyzed by PMI is the first committed step in the synthesis of Man-containing sugar chains and provides a link between glucose metabolism and mannosylation. The mannose activation is specifically crucial for the synthesis and organization of the cell wall and thus essential for survival of fungal species (Jin 2012). Indeed, studies with *A. nidulans and A. fumigatus* revealed that the activity of phophomannose isomerase 1 plays a central regulatory role in cell wall synthesis (Smith and Payton 1994; Fang et al. 2009). Rajesh et al. (2012) demonstrated that inactivation of the phophomannose isomerase gene in *Stretomyces coelicolor* also compromised cellular differentiation. As GPD-mannose is involved in the generation of mannosylated glycans, which are important cell wall components in bacteria and fungi, changes in the activity of phosphomannose isomerase can led to defects in cell wall biosynthesis. Although experiments with sugar transport were not performed in the present study we can consider that possibly a reduction of Glc-6P or Fru-6P could be related with the decrease in cell wall thickness and decrease of sugar content of cell walls when the *P. janczewskii* was grown on fructose (Figs. 3, 5).

Our findings provide evidences that fructose-containing sugars supplied as carbon source directly affect the cell metabolism of *P. janczewskii* thus explaining our previous results (Pessoni et al. 2005) on cell wall alteration when this fungus is cultured on inulin to induce extracellular inulinases.

In *A. nidulans* it was demonstrated that glucans are accumulated in the cell wall during vegetative growth and they serve as main reserve material required to massive cell proliferation at specific growth phases after glucose has been depleted from the medium (Zonneveld 1974). Therefore, the decrease in cell wall thickness followed by an increase in low molecular weight glucans found in the present study after carbon source depletion of the medium may be related to hydrolysis of cell wall components by glucanases. The glucanase secretion and subsequent utilization of their products could be an alternative way to supply the required carbon to maintain fungal metabolism at late stages of *P. janczewskii* development.

## Conclusion

In conclusion, our findings evidenced changes in the turgidity of the mycelium of *P. janczewskii*, which showed weak and collapsed hyphae when grown in medium containing fructose. This confirms our hypothesis that the effects of fructose polymers (inulin) on the cell wall are due to fructose itself. Moreover, our data show the induction of extracellular β-fructofuranosidases in *P. janczewskii* by fructose, explaining the production of such enzymes when inulin is used as carbon source.

Considering the importance of fructose-containing compounds produced by microorganisms to food industry and the recognition of the fungal cell wall as an ideal drug target, further studies in *P. janczewskii* related to the effect of fructose on cell wall remodeling and about the enzymes implicated in this process are pertinent and necessary.

## Methods

### Microorganism used

Strains of *P. janczewskii* Zaleskii (URM 3511, Universidade Federal de Pernambuco, Recife, Brazil) were obtained from the fungal collection of the Laboratory of Biodiversity at the Methodist University of São Paulo.

### Culture conditions

The fungus was grown in medium containing the following components (g L^−1^): NaNO_3_ (3), KH_2_PO_4_ (1), KCl (0.5), MgSO_4_·7H_2_O (0.5), FeSO_4_·7 H_2_O (0.01). Fructose, glucose or an equimolar mixture of glucose + fructose 30 mM were used as carbon sources. For the morphological and structural analyses, the fungus was grown on the same medium containing 1.5 % agar and sucrose 30 mM was used as control.

### Growth in solid medium

The growth curve of *P. janczewskii* was determined as described by Pessoni et al. (2005). Inocula of 5 mm in diameter obtained from pure cultures grown for 7 days on PDA were placed in the center of Petri dishes containing the culture media described above. The plates were incubated at 28 °C and the growth was evaluated by measuring the diameter of each colony at 3, 6, 9, 12 and 15 days. Data were obtained from four replicates.

### Light microscopy

The morphology of the hyphae of *P. janczew*skii was analyzed by light microscopy using slides prepared according to the microculture technique proposed by Lacaz-Ruiz (2000). Blocks (2 × 2 cm) of solid culture media containing different carbon sources (item 2) were added separately on microscope slides previously sterilized. Inocula (4 per block) of the fungus were added to the culture medium. Then each block was covered with a sterile coverslip. The slides were supported on a glass holder on moistened filter paper, inside in a Petri dish, all pre-sterilized. This set was incubated at 28 °C for 6, 9, 12 and 15 days. After each incubation period, coverslips were removed (five coverslips for each culture medium), mounted on slides, stained with cotton blue and examined under a light microscope (Nikon) for detailed observation of the hyphae. The images were captured by video camera (Motican) coupled to the microscope, and analyzed by the computer program AVSoft BioView 4. Three images were captured from each slide and from each image it was assessed the thickness of the hyphae, measuring ten different points. Data were subjected to analysis of variance (ANOVA) and *p* values <0.05 were compared by Tukey test.

### Scanning electron microscopy (SEM)

The hyphae of *P. janczewskii* grown on solid medium were analyzed by SEM. The samples were fixed in 2 % osmium tetroxide vapor for 24 h. The material was cut and deposited on a glass coverslip. Coverslips were placed in Petri dishes with moistened filter paper (moist chamber) containing a small open container with 2 % osmium tetroxide. The plates were wrapped with foil, leaving at room temperature for 24 h. After fixation, the material was kept in a chamber with silica for 3 h for drying and subsequently coated with a layer of gold for 39 s. The samples were examined and photographed in scanning electron microscope (Phillips).

### Growth in liquid medium

Discs of fungal culture were inoculated in Erlenmeyer flasks of 250 mL containing 100 mL of liquid culture medium with different carbon sources (item 2). The growth was monitored for 15 days at room temperature. At predetermined intervals, the mycelial mass was separated from the medium by filtration, washed with distilled water and dried and the dry matter determined. All tests were performed in triplicate.

### Quantification of sugars and proteins in the culture medium

For quantification of sugars in the liquid medium, the method of Somogyi (1945) was used and depending on the source of carbon glucose, fructose or equimolar mixture of glucose + fructose (100 μg mL^−1^) was used as standard. The protein content was determined by the method of Bradford (1976) using bovine serum albumin (BSA) as standard.

### Enzyme extraction

The extraction of extracellular enzymes was performed at defined points of the fungal growth curve according to Pessoni et al. (1999, 2007). After filtration, the liquid culture medium free from mycelial mass was centrifuged at 8000*g* for 15 min at 5 °C. The precipitate was discarded and the supernatant was filtered again in 0.45 mm Millipore filters, and the filtrate was considered as the crude enzyme extract.

### Assays of enzyme activity

The crude enzyme extract was assayed for enzyme activity using 1 % sucrose or inulin from *Helianthus tuberosus* (Sigma) as substrate. The activity was detected by quantification of released reducing sugars (Somogyi 1945), using fructose as standard for tests with inulin and an equimolar mixture of fructose and glucose, for sucrose. One enzyme unit (U) was defined as the amount of enzyme that releases 1 μmol fructose per minute, for tests with inulin or hydrolyzed 1 μmol of sucrose per minute, for tests with sucrose under the conditions tested. Except for some specific experiments, in which the conditions were mentioned, assays consisted of a mixture of 50 μL of enzyme extract and 50 μL of 1 % inulin or 1 % sucrose, incubated for 10 min at 55 °C, as described by Pessoni et al. (1999).

### Isolation of cell walls

At the end of each period of incubation in liquid medium, the culture media were filtered under vacuum through paper fiberglass. The mycelium was washed thoroughly with distilled water and frozen at −18 °C. Subsequently, the mycelium was resuspended in distilled water and cells disrupted by vortexing 3 times, 10 min each in an ice bath. The broken cells were separated by centrifugation at 8000*g* for 15 min and the waste sonicated for 1 h and centrifuged at 8000*g* for 15 min. The cell walls were lyophilized and stored in a desiccator. The disruption of the hyphae was monitored by light microscopy, using cotton blue staining.

### Quantification of sugars and proteins in the cell walls

One mg of lyophilized cell walls was resuspended in 1 mL of concentrated H_2_SO_4_ and total sugar content was determined by the phenol–sulfuric method (Dubois et al. 1956), using glucose as standard. One mg of lyophilized cell walls was resuspended in 1 mL of 1 N NaOH. The protein content was determined by the Bradford (1976) method using bovine serum albumin as standard.

### Extraction and quantification of chitin

Two mg of lyophilized cell walls were hydrolyzed with 6 N HCl at 90 °C for 48 h. After this period, the hydrolysates were filtered through glass fiber, concentrated to dryness in rotary evaporator at 50 °C and resuspended in deionized water (Nilsson and Bjurman 1998). The concentration of glucosamine in hydrolysates was determined colorimetrically using the method of Chen and Johnson (1983), modified. Aliquots of 1 mL of the hydrolyzed sample were added to 0.25 mL of a 4 % acetylacetone (4 % acetylacetone in 1.25 N sodium carbonate) and heated at 90° C for 1 h in sealed flasks. After cooling, 2 mL of ethanol were added and stirred to dissolve the precipitate formed. Then 0.25 mL Ehrlich’s reagent (1.6 g of *NN*-dimethyl-*p*-aminobenzaldehyde in 60 mL of a solution of ethanol: concentrated HCl 1:1) was added. The color formed was measured at 530 nm. A solution of glucosamine hydrochloride (100 µg mL^−1^) was used as standard.

### Fractionation and determination of molar mass

Fractionation of the cell walls was based on the methods of Domenech et al. (1996) and Carbonero et al. (2001). Samples of lyophilized cell walls were extracted with 1 M NaOH for 12 h at room temperature. After this period, the solubilized wall was centrifuged at 10,000*g* for 30 min at 20 °C. The supernatant was neutralized with HCl, dialyzed against distilled water and freeze-dried, and considered the Fraction I (FI), containing the water-soluble polysaccharides. This fraction was used to determine the apparent average molecular mass on a column by gel permeation chromatography. Aliquots of fraction I (FI) were dissolved in distilled water and centrifuged at 13,000*g* for 15 min at room temperature. The precipitates were discarded and the supernatants were applied on a glass column 1.5 × 120.0 cm (BIO-RAD) with Sepharose CL-6B resin (Pharmacia) equilibrated with 50 mM phosphate-citrate buffer, pH 5.2. The calibration of the column was performed with “Blue-dextran” to determine the empty volume of the column and dextrans of different molecular weights (10,000, 40,000 and 70,000 Da) as standards. Fractions of 2 mL were collected and monitored for carbohydrate content using the phenol–sulphuric acid method (Dubois et al. 1956).

## References

[CR1] Benthin S, Nielsen J, Villadsen J (1994). Galactose expulsion during lactose metabolism in *Lactococcus lactis* subsp. *cremoris* FD1 due to dephosphorylation of intracellular galactose 6-phosphate. Appl Environ Microbiol.

[CR2] Bradford MM (1976). A rapid and sensitive method for the quantitation of microgram quantities of protein utilizing the principle of protein-dye binding. Anal Biochem.

[CR3] Carbonero ER, Sassaki GL, Stuelp PM, Gorin PAJ, Woranovicz-Barreira SM, Iacomini M (2001). Comparative studies of the polysaccharides isolated from lichenized fungi of the genus *Cladonia*: significance as chemotypes. FEMS Microbiol Lett.

[CR4] Chaudhuri A, Bharadwaj G, Maheshwari R (1999). An unusual pattern of invertase activity development in the thermophilic fungus *Thermomyces lanuginosus*. FEMS Microbiol Lett.

[CR5] Chen GC, Johnson BR (1983). Improved colorimetric determination of cell wall chitin in wood decay fungi. Appl Microbiol Biotechnol.

[CR6] Costaglioli P, Meilhoc E, Jonatova I, Klein R, Masson J (1997). Secretion of invertase from *Schwanniomyces occidentalis*. Biotechnol Lett.

[CR7] Damveld RA, Arentshorst M, Franken A, vanKuyk PA, Klis FM, van den Hondel CA, Ram AF (2005). The *Aspergillus niger* MADS-box transcription factor RlmA is required for cell wall reinforcement in response to cell wall stress. Mol Microbiol.

[CR8] de Nobel JG, Ruiz C, Martin H, Morris W, Brul S, Molina M, Klis FM (2000). Cell wall perturbation in yeast results in dual phosphorylation of the Slt2}Mpk1 MAP kinase and in an Slt2-mediated increase in FKS2-lacZ expression, glucanase resistance and thermotolerance. Microbiology.

[CR9] Dhake AB, Patil MB (2007). Effect of substrate feeding on production of fructosyltransferase by *Penicillium purpurogenum*. Braz J Microbiol.

[CR10] Doehlemann G, Molitor F, Hahn M (2005). Molecular and functional characterization of a fructose specific transporter from the gray mold fungus *Botrytis cinerea*. Fungal Genet Biol.

[CR11] Domenech J, Barasoain I, Prieto A, Gómez-Miranda B, Bernabé M, Leal JA (1996). An antigenic water-soluble glucogalactomannan extracted from cell walls of *Paecilomyces fumosoroseus* and *Paecilomyces farinosus*. Microbiology.

[CR12] Dubois M, Gilles KA, Hamilton JK, Rebers PA, Smith F (1956). Colorimetric method for determination of sugars and related substances. Anal Chem.

[CR13] Dynesen J, Smits HP, Olsson L, Nielsen J (1998). Carbon catabolite repression of invertase during batch cultivations of *Saccharomyces cerevisiae*: the role of glucose, fructose, and mannose. Appl Microbiol Biotechnol.

[CR14] Fang W, Yu X, Wang B, Zhou H, Ouyang H, Ming J, Jin C (2009). Characterization of the *Aspergillus fumigatus* phosphomannose isomerase Pmi1 and its impact on cell wall synthesis and morphogenesis. Microbiology.

[CR15] Flipphi M, Sun J, Robellet X, Karaffad L, Fekete E, Zeng AP, Kubicek CP (2009). Biodiversity and evolution of primary carbon metabolism in *Aspergillus nidulans* and other *Aspergillus* spp. Fungal Genet Biol.

[CR16] Fontaine T, Mouyna I, Harthand RP, Paris S, Latgé JP (1997). From the surface to the inner layer of the fungal cell wall. Biochem Soc Trans.

[CR17] Galeote V, Novo M, Salema-Oom M, Brion C, Valério E, Gonçalves P, Dequin S (2010). FSY1, a horizontally transferred gene in the *Saccharomyces cerevisiae* EC1118 wine yeast strain, encodes a high-affinity fructose/H+ symporter. Microbiology.

[CR18] Ghfir B, Fonvieille JL, Dargent R (1997). Influence of essential oil of *Hyssopus officinalis* on the chemical composition of the walls of *Aspergillus fumigatus* (Fresenius). Mycopathologia.

[CR19] Grimm LH, Kelly S, Krull R, Hempel DC (2005). Morphology and productivity of filamentous fungi. Appl Microbiol Biotechnol.

[CR20] Jaeken J, Pirard M, Adamowicz M, Pronicka E, Schaftingen EV (1996). Inhibition of phosphomannose isomerase by fructose 1-phosphate: an explanation for defective *N*-glycosylation in hereditary fructose intolerance. Pediatr Res.

[CR21] Jin C (2012). Protein glycosylation in *Aspergillus fumigatus* is essential for cell wall synthesis and serves as a promising. Int J Microbiol.

[CR22] Lacaz-Ruiz R (2000). Manual Prático de Microbiologia Básica.

[CR23] Latgé JP (2010). Tasting the fungal cell wall. Cell Microbiol.

[CR24] Liu H, Zeng Z, Wang P, Gong G, Wang L, Zhao G (2013). Morphological changes induced by class III chitin synthase gene silencing could enhance penicillin production of *Penicillium chrysogenum*. Appl Microbiol Biotechnol.

[CR25] Mellado EG, Dubreucq PM, Sarfati J, Paris S, Diaquin M, Holden DW, Rodriguez-Tudela JL, Latgé JP (2003). Cell wall biogenesis in a double chitin synthase mutant (chsG-;chsE-) of *Aspergillus fumigatus*. Fungal Genet Biol.

[CR26] Moriyama S, Muguruma M, Ohta K (2006). Quantitative expression analysis of inulinase gene cluster of *Penicillium* sp. strain TN-88. J Biosci Bioeng.

[CR27] Nilsson K, Bjurman J (1998). Chitin as an indicator of the biomass of two wood-decay fungi in relation to temperature, incubation time, and media composition. Can J Microbiol.

[CR28] Pessoni RAB, Figueiredo-Ribeiro RCL, Braga MR (1999). Extracelullar inulinases from *Penicillium janczewskii*, a fungus isolated from the rhizosphere of *Vernonia herbacea* (Asteraceae). Appl Microbiol Biotechnol.

[CR29] Pessoni RAB, Freshour G, Figueiredo-Ribeiro RCL, Hahn MG, Braga MR (2005). Cell-wall structure and composition of *Penicillium janczewskii* as affected by inulin. Mycologia.

[CR30] Pessoni RAB, Braga MR, Figueiredo-Ribeiro RCL (2007). Purification and properties of exo-inulinases from *Penicillium janczewskii* growing on distinct carbon sources. Mycologia.

[CR31] Rajesh T, Song E, Kim JN, Le BR, Kim EJ, Park SH, Kim YG, Yoo D, Park HY, Choi YH, Kim BG, Yang YH (2012). Inactivation of phosphomannose isomerase gene abolishes sporulation and antibiotic production in *Streptomyces coelicolor*. Appl Microbiol Biotechnol.

[CR32] Rand JB, Tatum EL (1980). Fructose transport in *Neurospora crassa*. J Bacteriol.

[CR33] Romero-Gómez S, Augur C, Viniegra-Gonzáles G (2000). Invertase production by *Aspergillus niger* in submerged solid-state fermentation. Biotechnol Lett.

[CR34] Rubio MC, Maldonado MC, Aznar PY, Navarro AR (2003). Producción y caracterización de una invertasa extracelular de *Penicillium glabrum*. Aliment Latinoam.

[CR35] Ruiz-Herrera J (1992). Fungal cell wall: structure, synthesis, and assembly.

[CR36] Ruiz-Herrera J, Ortiz-Castellanos L, Martínez AL, León-Ramírez C, Sentandre R (2008). Analysis of the proteins involved in the structure and synthesis of the cell wall of *Ustilago maydis*. Fungal Genet Biol.

[CR37] Santamaría RI, Leal F, Díaz M, Fernández-Abalos JM (2002). Morphological and physiological changes in *Streptomyces lividans* induced by different yeasts. Arch Microbiol.

[CR38] Santos A, Marquina D, Leal JA, Peinado JM (2000). (1-6)-β-d-glucan as cell wall receptor form *Pichia membranifaciens* killer toxin. Appl Environ Microbiol.

[CR39] Seidl V (2008). Chitinases of filamentous fungi: a large group of diverse proteins with multiple physiological functions. Fungal Biol Rev.

[CR40] Silva MLC, Martinez PF, Izeli NF, Silva IR, Vasconcelos AFD, Cardoso MS, Stelutti RM, Giese EC, Barbosa AM (2006). Caracterização química de glucanas fúngicas e suas aplicações biotecnológicas. Quím Nova.

[CR41] Smith DJ, Payton MA (1994). Hyphal tip extension in *Aspergillus nidulans* requires the *manA* gene, which encodes phosphomannose isomerase. Mol Cell Biol.

[CR42] Somogyi M (1945). A new reagent for the determination of sugars. J Biol Chem.

[CR43] Wei H, Vienken K, Webera R, Buntinga S, Requeña N, Fischer R (2004). A putative high affinity hexose transporter, hxtA, of *Aspergillus nidulans* is induced in vegetative hyphae upon starvation and in ascogenous hyphae during cleistothecium formation. Fungal Genet Biol.

[CR44] Yuan XL, Goosen C, Kools H, Van der Maarel MJ, Van den Hondel CA, Dijkhuizen L, Ram AF (2006). Database mining and transcriptional analysis of genes encoding inulin-modifying enzymes of *Aspergillus niger*. Microbiology.

[CR45] Yuan XL, Roubos JA, van den Hondel CA, Ram AF (2008). Identification of InuR, a new Zn(II)2Cys6 transcriptional activator involved in the regulation of inulinolytic genes in *Aspergillus niger*. Mol Genet Genomics.

[CR46] Zonneveld BJM (1974). α-1,3 Glucan synthesis is correlated with α-1,3 glucanase synthesis, condition and fructification in morphogenetic mutants of *Aspergillus nidulans*. J Gen Microbiol.

